# Effect of insecticide-treated bed net usage on under-five mortality in northern Ghana

**DOI:** 10.1186/s12936-015-0827-8

**Published:** 2015-08-07

**Authors:** Clifford Afoakwah, Jacob Nunoo, Francis Kwaw Andoh

**Affiliations:** Department of Economics, University of Ghana, Legon-Accra, Ghana; Department of Economics, University of Cape Coast, Cape Coast, Ghana

**Keywords:** ITN usage, Under five mortality, Health facility delivery, Northern Ghana

## Abstract

**Background:**

Although under-five mortality rate seems to be declining in Ghana, the northern part of the country has higher levels of under-five mortality vis-à-vis the national rates. This research examines the correlates of the high under-five mortality among children in the northern part of Ghana, with emphasis on the usage of insecticide-treated bed net (ITN), as recommended by the World Health Organization.

**Methods:**

A total of 3,839 under-five children sourced from the Ghana Demographic and Health Survey—was used for this study. Univariate descriptive statistics was employed to describe the variables used for the empirical estimation. The maximum likelihood estimation technique was used to estimate a logit model in other to determine the effect of insecticide treated bed net usage on under-five mortality.

**Results:**

Insecticide-treated bed net usage among children enhances their survival rates. Thus, under-five mortality among children who sleep under treated bed nets is about 18.8% lower than among children who do not sleep under treated bed nets. While health facility delivery was found to reduce to reduce under-five mortality, child bearing among older women is detrimental to the survival of the child.

**Conclusions:**

The study, therefore, recommends that policies targeting reduction in under-five mortality in northern Ghana should consider not mere availability of ITNs in the household, but advocate the usage of these treated nets. The study recommends to the Ministry of Health to extend their services to unreached rural communities to encourage health facility delivery to reduce under-five mortality.

## Background

Reducing under-five mortality rate remains a major concern for countries especially the developing countries. A lower under-five mortality rate is an indication of an improved child well-being as well as the coverage and success of child survival intervention programmes. The Millennium Development Goal 4 (MDG 4) specifically draws the world’s attention to the need reduce under-five mortality rate by two-thirds between 1990 and 2015. The World Health Organization (WHO) reported that about 6.3 million children under age five died worldwide in 2013, while about 17,000 under-five children die every day [1].

Although there is a declining trend in the national under-five mortality rate in Ghana, the three northern regions—Northern, Upper East and Upper West regions—still experience a high incidence of under-five death. In 2003, while the national average of under-five mortality was estimated to be 111 deaths per 1,000 live births, that of the Northern, Upper East and Upper West were estimated to be 154, 79, and 154, respectively. Moreover, while the 2008 demographic and health survey (DHS) indicated a significant reduction in under-five mortality rate of 80 per 1,000 live births, the northern, Upper East, and Upper West regions were recorded as 137, 79, and 142, respectively [2, 3].

Malaria accounted for 35% of deaths in children below the age of 5 years. Each year, between 3.1 and 3.5 million cases of clinical malaria are reported in public health facilities, of which 900,000 cases are children under-five. Indeed, malaria is a major cause of severe anaemia, among under-five children. The high incidence of the disease is owed to the widespread of the most effective malaria vector *Anopheles gambiae*, which is also one of the most difficult to control. *Plasmodium falciparum*, which is the malaria parasite that causes a greater percentage of severe clinical forms, is the most widespread in Africa [4].

Insecticide-treated mosquito nets (ITNs) have emerged as one of the three primary interventions recommended by the World Health Organization Global Malaria Programme (WHO/GMP) for effective malaria control. A recent study has shown that about 60% of all adults and children can achieve equitable community-wide benefits of the use of ITNs in a community [5]. ITNs work as a vector control intervention for reducing malaria transmission and other infections transmitted via insects. Although the use of ITNs is considered to be a key primary health intervention for preventing malaria, a leading cause of under-five mortality, it is still not clear if its usage has had any effect on under-five mortality in the three northern regions of Ghana. The objective of this paper was to empirically estimate the extent of the effect of the usage of ITN on under-five mortality in the three northern regions of Ghana.

## Empirical studies on ITN usage and health outcomes

Lim et al. [6] investigated the associations between ITNs and health in sub-Saharan Africa. Their finding indicates that the recent scale-up in ITN coverage in several sub-Saharan African countries has been accompanied by significant reductions in child mortality. The study further notes that additional health gains could be achieved with further increases in ITN coverage in populations at risk of malaria. Much as the study provides relevant policy orientation, it has been criticized on the grounds that the methodology applied in establishing the association between ITNs and child mortality does not disentangle country specific effect from pooled countries effect. Fullman et al. [7] assessed the effectiveness of ITNs and indoor residual spraying (IRS) in reducing malaria morbidity and child mortality in sub-Saharan Africa. They found greater reductions in malaria morbidity while positive health gains were recorded among children who combined ITNs with IRS.

Akachi and Atun [8] combined multiple data sources and used panel data regression analysis to study the relationship among investment, service delivery/intervention coverage, and impact on child health by observing changes in 34 sub-Saharan African countries over the years 2002–2008. Impact of ITN/IRS coverage on under-five mortality was significant in reducing child deaths in sub-Saharan Africa. Loha et al. [9] used a discrete Poisson-based model to study the effect of bed nets and IRS on spatio-temporal clustering of malaria in a village in South Ethiopia. It was found that free mass distribution of ITNs did not influence the spatio-temporal clustering of malaria, but eliminated malaria clustering. Komazawa et al. [10] investigated the effectiveness of long-lasting insecticidal nets for preventing childhood deaths among non-net users in Western Kenya. Using the database from Mbita Health and Demographic Surveillance System (HDSS), children younger than age five were assessed over four survey periods. The results from the Cox proportional hazard models showed that current LLIN coverage level (about 35%) could induce a community effect to protect children sleeping without bed nets even in a malaria-endemic area, a better system is needed to monitor the current malaria situation globally in order to optimize malaria control programmes with limited resources.

Peña et al. [11], in a cohort analysis of infant survival based on reproductive histories of a representative sample of 10,867 women aged 15–49 years in León, Nicaragua, found that both young and older maternal age, as well as high parity, was linked to higher infant mortality risks. Also, boys had a higher mortality rate than girls and infants whose mothers had higher education had a lower risk of dying.

The conclusion from the empirical studies suggests that although few studies found otherwise, there is a consensus on the efficacy of ITN usage in the prevention of malaria and hence in reducing under-five mortality.

## Methods

### Model specification

This study assumes that the mother derives utility from the good health of the child and that there is disutility to the parent, in the form of pain, when a child dies. This means parents make informed choices over their life cycle to improve the survival of their children through investment in health care (preventive or curative health care). Thus, it is reasoned that the decision of ensuring good health for the child is the responsibility of the parents (see [12, 13]).

The probability that an infant survives the first 5 years of life (*π*) is a function of utilization of ITNs (*I*_*TN*_) and other factors such as birth order of the child (*B*^*c*^), health facility delivery (*H*^*f*^), household size (*h*^*z*^), as well as exogenous risk and productive efficiency factors such as parents education (*E*^*m*^). Based on the Grossman model [14], both father’s and mother’s education are included as important factors that determine demand for preventive healthcare. The survival probability production function is thus specified as:

1 $$\pi = \delta (I_{TN} ,B^{c} ,H^{f} ,h^{z} ,E^{m} ,X)$$

where *π* is the probability that the child survives the first 60 months after births and X is a vector of other exogenous variables such as regional dummies and urban dummy. The probability of child death (1 − *π*) is considered as the dependent variable since the focus of this paper is on under-five mortality rather than under-five survival. The corresponding empirical logit model is:

2 $$(1 - \pi )_{i}^{*} = {\mathbf{z}}_{i} + \eta _{i} {\text{ with (1}} - \pi )_{i} = \left\{ {\begin{array}{*{20}l} {1\quad if(1 - \pi )_{i}^{*} > 0} \\ {0\quad otherwise,} \\ \end{array} } \right.$$

where (1 − *π*)is the probability that a child dies before age five, **z**_*i*_ is a vector of exogenous factors influencing under-five mortality; **β** is a vector of unknown parameters; and *η*_*i*_ is an error term with mean zero and variance of *σ*_*η*_^2^. It also capture unobserved factors, which influence demand for each of the health inputs and outputs, which encompass not only tastes but also innate healthiness and other individual characteristics. The maximum likelihood estimation technique is employed. This estimation technique is deemed appropriate because equation 5 satisfies the assumptions underlying the MLE, key of such assumptions is that the estimable variable (child death) is dichotomous [15].

### Data source

Data for this study was sourced from Ghana Demography and Health Survey (GDHS 2008). The GDHS 2008 is a national survey covering all ten regions of the country. The survey was designed to collect, analyse, and disseminate information on housing and household characteristics, education, maternal health and child health, nutrition, family planning, gender, and other health related issues. GDHS 2008 is the fifth DHS survey to be undertaken in Ghana since 1988. All five surveys have been designed and administered by Ghana Statistical Service of Ghana, the official national agency responsible for data collection, archiving and management.

## Results and discussion

### Descriptive statistics

Table 1 presents descriptive statistics of variables used in the regression analysis. The percentage of children that died before age five in the northern region prior to the study was 14.8 while the average child was the third of his/her parent children. The average year of schooling for the mother of the child is 1 year while that of the husband was 7 years. The study showed that about 76% of these women have no education, a situation which is very intriguing and needs attention. While 52.1% of children used no net for sleeping, 23.1 and 24.2% used only treated net and only untreated net for sleeping, respectively. This implies children in northern Ghana do not only lack access to ITN, but about 24% of these children use untreated bed nets. The average number of members in a northern household is about seven individuals. In terms of health facility delivery as a proxy for skilled birth attendant, about 9.3% of children were delivered at a health facility. As at 2009 (a year after the survey), the northern part had about 579 health facilities including teaching hospitals, regional hospitals, psychiatric hospitals, polyclinics, health care centres and clinics, as well as maternity homes and CHIP compounds. The northern region alone has about 300 whiles the Upper East and Upper West regions have 144 and 135 respectively, of these health facilities [2]. The average age of a mother in these regions is 38 years, an indication that most of these women are in their prime ages. In terms of poverty, about 64.5% of households in these three regions in northern Ghana are categorized as poorest, while 3% are defined as richest.

**Table 1:** Descriptive statistics of determinants of under-five mortality in Ghana

Explanatory variable	Variables description	Mean	SD
Under-five death	Whether the child died before age 5	0.148	0.355
Birth order	Childs birth order	3.305	2.166
Mother’s education	Years of schooling of the mother	1.238	3.024
Male	Male child	0.514	0.5
Upper East	Whether the child is in the Upper East region	0.407	0.491
Northern	Whether the child is in the Northern region	0.264	0.441
Upper West	Whether the child is in the Upper West region	0.328	0.47
Partner’s education	Husbands years of schooling	7.424	21.617
Female head	Female headed household	0.163	0.37
No net	Whether child use no net for sleeping	0.521	0.499
Only treated net	Whether child use only treated net for sleeping	0.231	0.422
Only untreated net	Whether child use only untreated net for sleeping	0.242	0.432
Household size	Total household members	7.324	3.337
Urban	Whether the child lives in an urban area	0.177	0.382
Health facility	Whether the child was delivered at a health facility	0.093	0.29
Mothers age	Age of the mother	36.677	7.579
Poorest	Whether the child belongs to a poorest household	0.645	0.478
Poorer	Whether the child belongs to a poorer household	0.175	0.380
Middle	Whether the child belongs to a middle wealth household	0.090	0.286
Richer	Whether the child belongs to a richer household	0.059	0.236
Richest	Whether the child belongs to a richest household	0.030	0.170

### Regression analysis

In order to ascertain the correct specification of the model and also the how well the model fit the data available (reliability of the model), a Link test (test for specification) and a Hosmer–Lemeshow test were respectively performed prior to the regression analyses. The p-value of the square of the explained sum of square of the link test was 0.432, thus, failing to reject the null hypothesis that the logit model has no omitted variables. The results of the Hosmer–Lemeshow test which assesses whether or not the observed event rates match expected event rates in subgroups of the model population for binary regression models [16], showed a p-value of 0.251 indicating that the null hypothesis cannot be rejected. Hence, the specified logit model is fit and therefore reliable. This is shown on Table 2. In addition, the Chi square statistic indicates that the regression line is a good fit at 1% significance level.

**Table 2:** Logit regression of determinants under-five mortality in Ghana

Explanatory variables	Coefficient	Odds ratio
Birth order	−0.079 (0.025)***	0.924
Mother’s education (in years)	−0.016 (0.019)	
Male (1/0)	0.177 (0.093)*	1.193
Region (base = Upper East)		
Northern	0.300 (0.128)***	1.357
Upper West	0.405 (0.129)***	1.506
Father’s education (in years)	−0.0003 (0.002)	
Female household head (1/0)	−0.061 (0.137)	
Bed net use (base = no net)		
Only treated nets	−0.209 (0.094)**	0.812
Only untreated nets	0.041 (0.115)	
Household size	−0.028 (0.016)*	0.978
Urban (1/0)	0.286 (0.286)*	1.302
Health facility (1/0)	−0.343 (0.208)*	0.713
Mother’s age (in years)	0.040 (0.007)***	1.041
Wealth index (base = poorest)		
Poorer	−0.407 (0.136)***	0.665
Middle	−0.591 (0.207)***	0.560
Richer	−0.234 (0.226)	
Richest	−0.730 (0.377)*	0.478
Constant	−3.155 (0.334)***	
N	3,839	
Pseudo R^2^	0.028	
Prob > χ^2^	91.14***	
Goodness of fit test (p-value)	0.465	
Link test (y_hat) (y-hatsq)	0.000 0.432	

*Source* Own computation.

Robust standard errors are in parentheses.

*, **, *** Significant at 10, 5 and 1%, respectively.

The regression results show that the usage of only untreated nets increases the likelihood that a child will die before age five. However, it is not statistically significant. The variable of interest, the usage of treated bed nets is found to be statistically significant at 5% in reduce the likelihood of a child dyeing before he/she turns 5 years. The odds ratio 0.812 implies that under-five mortality among children who sleep under treated bed nets is 18.8% lower than among children who do not sleep under treated bed nets. This result makes sense in that if children sleep under treated bed nets their survival rate increases, thereby reducing their probability of dying before age five. It can be reasoned that children who sleep under ITN are not predisposed to mosquitoes hence the risk of contracting malaria is reduced. This finding confirms the study by Fullman et al. [7] who observed a significant risk reduction against parasitaemia in medium and high transmission associated with living in households with both ITNs and IRS in sub-Saharan Africa. Their assessment showed a greater reduction in malaria morbidity while positive health gains for children were achieved with ITNs and IRS. ITNs have been shown to avert an estimated 50% of malaria cases, making protective efficacy significantly higher than that of untreated nets in Ghana.

Compared to females, male children were more likely to die before age five. This variable was significant at 10% significance level. Under-five mortality among male children is about 19.35 more than among female children in northern Ghana. This corroborates the findings by Peña et al. [11] in Nicaragua. It has been noted that male children have relatively weak immune system which in turns increases their risk of dying compared to female children, all other things being equal. Household size is also found to have a statistically significant negative effect on under-five mortality. The level of significance is 10%. The effect of each member in the household on increasing the survival rate of an under-five child in the household is about 2.2%. This suggests that in the northern part of Ghana household members provide support towards the well-being of children in household. It is the case where the welfare of children is considered as the responsibility of all in society. Thus, children benefit from a form of social capital which is very key to the survival of the child during this formative period. In some instances, household members provide financial support, food and security towards the upkeep of the child.

Health facility delivery is found to be a very key predictor of under-five mortality in northern Ghana. Significant at 10%, children delivered at health facility, where they were delivered by a skilled birth attendant, are less likely to die before age five. Thus, under-five mortality among children delivered at health facility is about 28.7% lower than among children who were not delivered at health facility. This finding emphasizes the importance of skilled birth attendants in child health. Skilled birth attendants are among able to detect any immediate complications emanating from the delivery process, which might affect the child survival. Early treatment is given to children who are detected with any complication in order to increase their survival rates. This finding is in line with the report by Hobcraft [17] that usage of medical facilities reduces child deaths.

The order of birth of the child at 1% significantly determines under-five mortality in northern Ghana. Children with older siblings are less likely to die before age five. Thus, mortality among children who have older siblings is about 7.6% low. This suggests that the principle learning by doing may also apply in child bearing. As a woman continues to give birth, she gains experience in child upbringing. Subsequent children, therefore, become beneficiaries of this experience in terms of quality of childcare. This finding is consistent with the Ghanaian cultural setting where women are mostly in charge of child upbringing with some or no support from the husband. The kind of experience that these women gain as a result of childbirth includes postnatal treatment, early breastfeeding, and early detection of unusual child infections. Although childbirth gives the woman the necessary experience to take good care of subsequent deliveries, childbirth among older women is not encouraged, because it increases the child’s likelihood of dying before age five. This variable is significant at 1% significance level. This finding supports the study by Peña et al. [11] who concluded that older maternal age, was linked to higher infant mortality risks.

Finally, household wealth plays a vital role in guaranteeing child survival in northern Ghana. Children born to well to do families have greater chance of living beyond age five vis-à-vis children born to poor families. At 10% significance level, under-five mortality among children from categorized richest families is about 52.2% lower than among children from households categorized as poorest. This however is not surprising since the rich is able to purchase health production inputs to improve on their children’s health stock.

## Conclusions

This paper has tested the hypothesis that usage of ITN among children in northern Ghana can reduce under-five mortality. The main finding of this paper seem to support similar studies in sub-Saharan African as well as other developing economies, like Nicaragua, thus, ITN usage reduces under-five mortality. Other findings from this paper are the fact that child bearing among older mothers can impact negatively on the child’s survival, health facility delivery helps to reduce under-five mortality. Based on the findings of the study, it is recommended that global and national efforts for scaling up the coverage of ITNs as a key child survival intervention should be needs to be strengthened in northern Ghana. These efforts need to be sustained and improved in order to reduce the high rate of under-five mortality in the northern hemisphere of Ghana. This will contribute toward achieving under-five mortality target 40 deaths per 1,000 live births in Ghana by the end of 2015 (MDG 4).

## Appendix

Parents’ utility is conceptualized to be a function of a number of surviving children (C) and other consumption goods (G).

3 $$U = u(C,G).$$

The number of children is endogenous because it depends on the survival probability production function of the children, *π* which in turn depends upon such endogenous inputs as the quantity and quality of medical care, nutrition, and the use of ITNs. Since the study focuses on the use of ITNs, the probability that an infant survives the first 5 years of life (*π*) is specified as a function of utilization of ITNs (*I*_*TN*_), and other covariates only holding all others constant;

4 $$\pi = \delta (I_{TN} ,B^{c} ,H^{f} ,h^{z} ,E^{m} ,X).$$

The survival of the child is said to be a function of a vector of other factors such as birth order of the child (*B*^*c*^), health facility delivery (*H*^*f*^), household size (*h*^*z*^), as well as exogenous risk and productive efficiency factors such as parents education (*E*^*m*^). In this study, both father’s education and mother’s education were included in the model. X is a vector of other exogenous variables such as regional dummies and urban dummy. Equation (4) is the survival probability production function. The use of ITNs has been confirmed to reduce the incidence of malaria which is the principal cause of deaths among children under 5 years [6, 8, 10]. Since the focus of this paper is on under-five mortality, we measure the probability of under-five death as (1 − *π*).

The parents maximize their utility function subject to the budget constraint of income specified in Eq. (5) as:

5 $$P_{TN} I_{TN} + P_{G} G = W$$

In Eq. 5, *P*_*G*_ is the price of consumption goods not related to the production of health, *W* is the income of the household which is made up of both labour and non-labour income. Implicit in Eq. 5 is the time constraint comprising of time allocated to the production of health, leisure and labour supply, which is equal to total time endowment. Household income is proxied by wealth index of the household due to lack of information on household income in our dataset. *P*_*TN*_ is the price of treated bed net. It is however normalized to one for all households because the bed nets are distributed for free to mothers.

Finally, by substituting Eq. (4) into Eq. (3) and maximizing subject to the budget constraint in Eq. (5) gives Eq. (6), which presents the demand for child health by the parent, or in other words, a child health production function:

6 $$\pi = \delta (P_{G} ,A,W)$$

In Eq. 6, *π* measure the probability that the child lives 60 months and beyond; which is a function of price of consumption goods (P_G_), socio-demographic characteristics (A) defined earlier, and income (W).
